# Supplementary material for: Self-current induced spin-orbit torque in FeMn/Pt multilayers
Source: Sci Rep. 2016 May 17;6:26180. doi: 10.1038/srep26180 (PMC4868966; doi:10.1038/srep26180)
Supplement: Supplementary Information [file srep26180-s1.pdf]

Self-current induced spin-orbit torque in FeMn/Pt multilayers

Yanjun Xu[1,3], Yumeng Yang[1,2], Kui Yao[2], Baoxi Xu[3], and Yihong Wu[1*]

[1]*Department of Electrical and Computer Engineering, National University of Singapore, 4 Engineering Drive 3, Singapore 117583, Singapore*

[2]*Institute of Materials Research and Engineering, A*STAR (Agency for Science, Technology and Research), 2 Fusionopolis Way, 08-03 Innovis, Singapore 138634, Singapore*

[3]*Data Storage Institute, A*STAR (Agency for Science, Technology and Research), 2 Fusionopolis Way, 08-01 Innovis, Singapore 138634, Singapore*

*Author to whom correspondence should be addressed: elewuyh@nus.edu.sg

Supplementary Figures

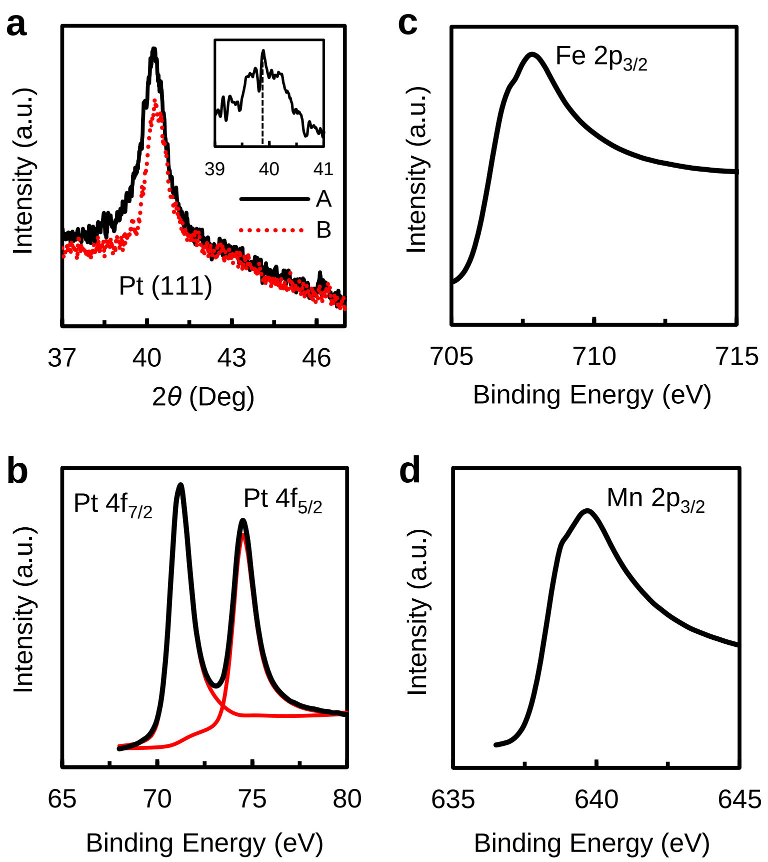

Supplementary Figure S1: Structural properties. (**a**) XRD patterns of the multilayer samples: Pt(3)/[FeMn(0.6)/Pt(0.6)]$_{20}$ (Curve A) and [FeMn(0.6)/Pt(0.6)]$_{20}$ (Curve B), covering the range of bulk fcc Pt (111) peak at 39.8° and bulk fcc FeMn (111) peak at 43.5°, using the Cu K_α radiation. Inset of (a): intensity difference between A and B (A-B). (**b-d**) XPS spectra of the Pt(3)/[FeMn(0.6)/Pt(0.6)]$_{20}$ sample.

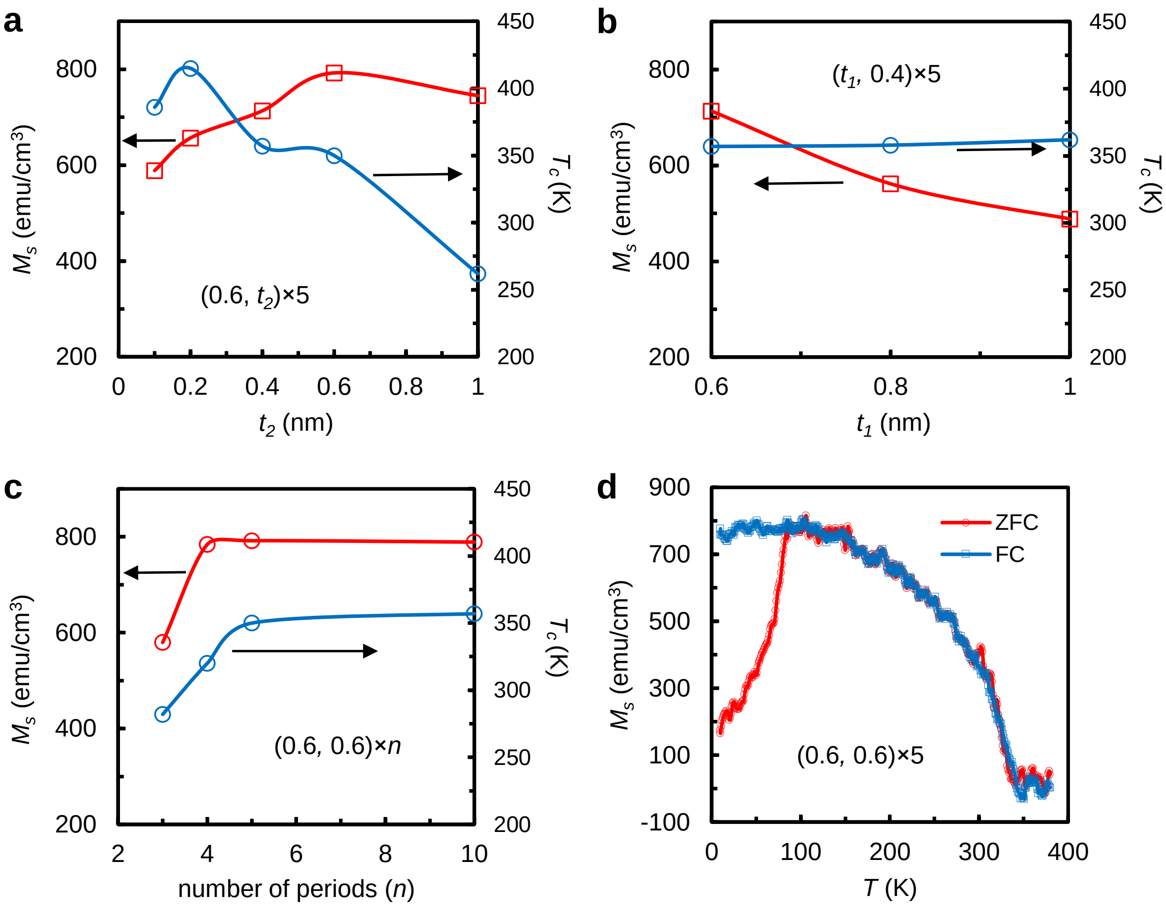

Supplementary Figure S2: Layer thickness and periodicity dependence of magnetic properties. (**a**) T_C and M_s (at 50 K) as a function of t_2 for (0.6, t_2)×5 samples. (**b**) T_C and M_s (at 50 K) as a function of t_1 for (t_1, 0.4)×5 samples. (**c**) T_C and M_s (at 50 K) as a function of n for (0.6, 0.6)×n samples. All the data are extracted from the *M*-*T* curves in Fig. 1b,c,d of the main text. The legend *(*t_1*,* t_2*)*×n denotes a multilayer with a FeMn thickness of t_1, Pt thickness of t_2, and a period of n. (**d**) ZFC (in red) and FC (in blue) curves for Pt(3)/[FeMn(0.6)/Pt(0.6)]$_5$, measured from 10 K to 380 K with an in-plane field of 100 Oe.

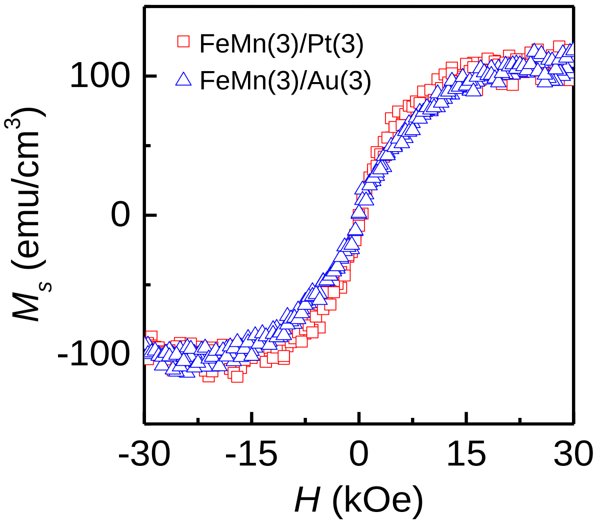

Supplementary Figure S3: Comparison of *M-H* curves between FeMn(3)/Au(3) and FeMn(3)/Pt(3) bilayers. The magnetic moment of both samples was measured by sweeping an in-plane field from -3 T to +3 T. The magnetization (M_s) is obtained by dividing the total moment over the volume of FeMn layer, and then plotted against the sweeping field. The difference in magnetization between two samples is negligible.

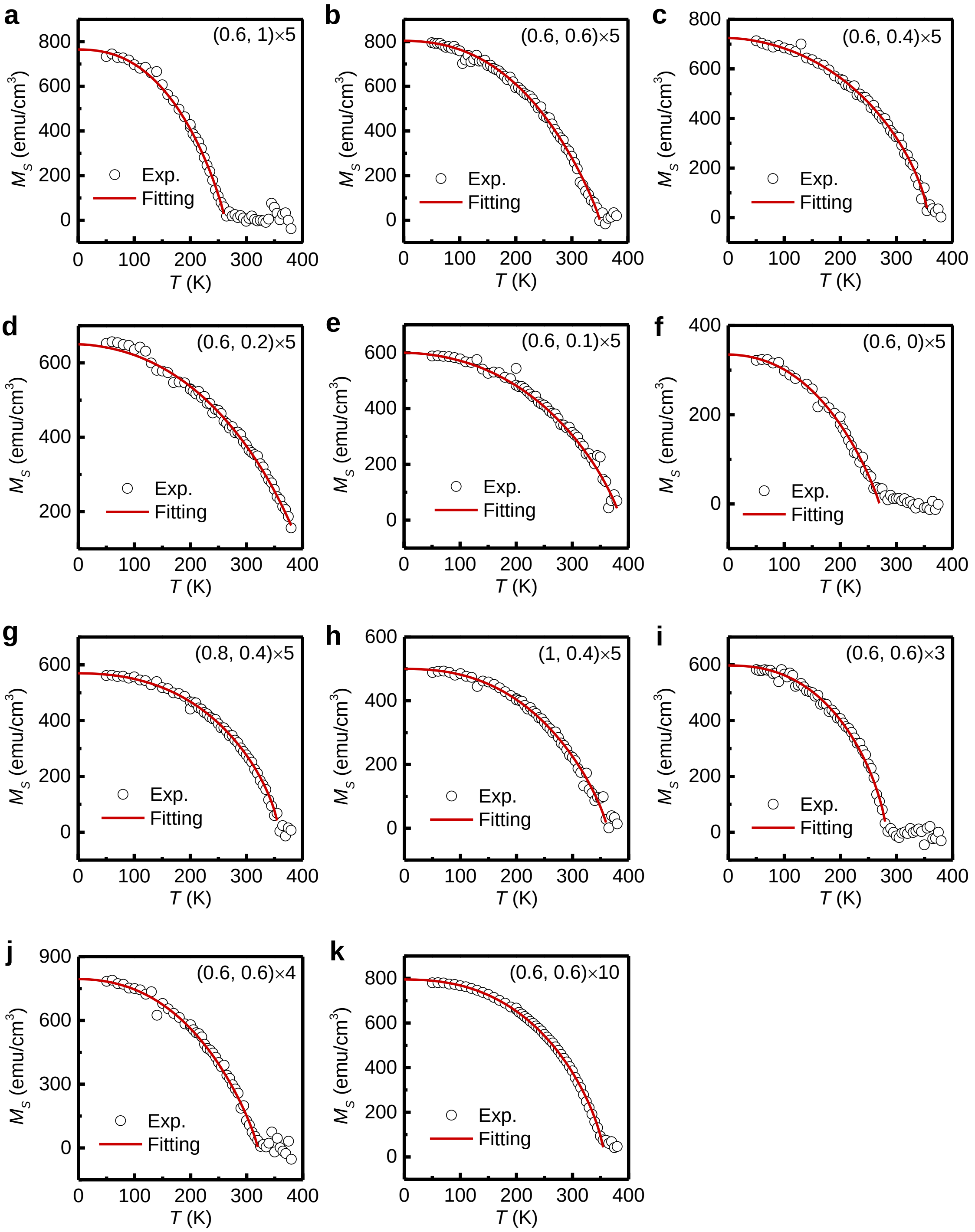

Supplementary Figure S4: Experimental (open circle) and fitted (solid line) *M-T* curves. The experimental dada are the same as those shown in Fig. 1 of the main text. Fitting is performed using Supplementary Equation (S9), and the fitting parameters are listed in Supplementary Table S1.

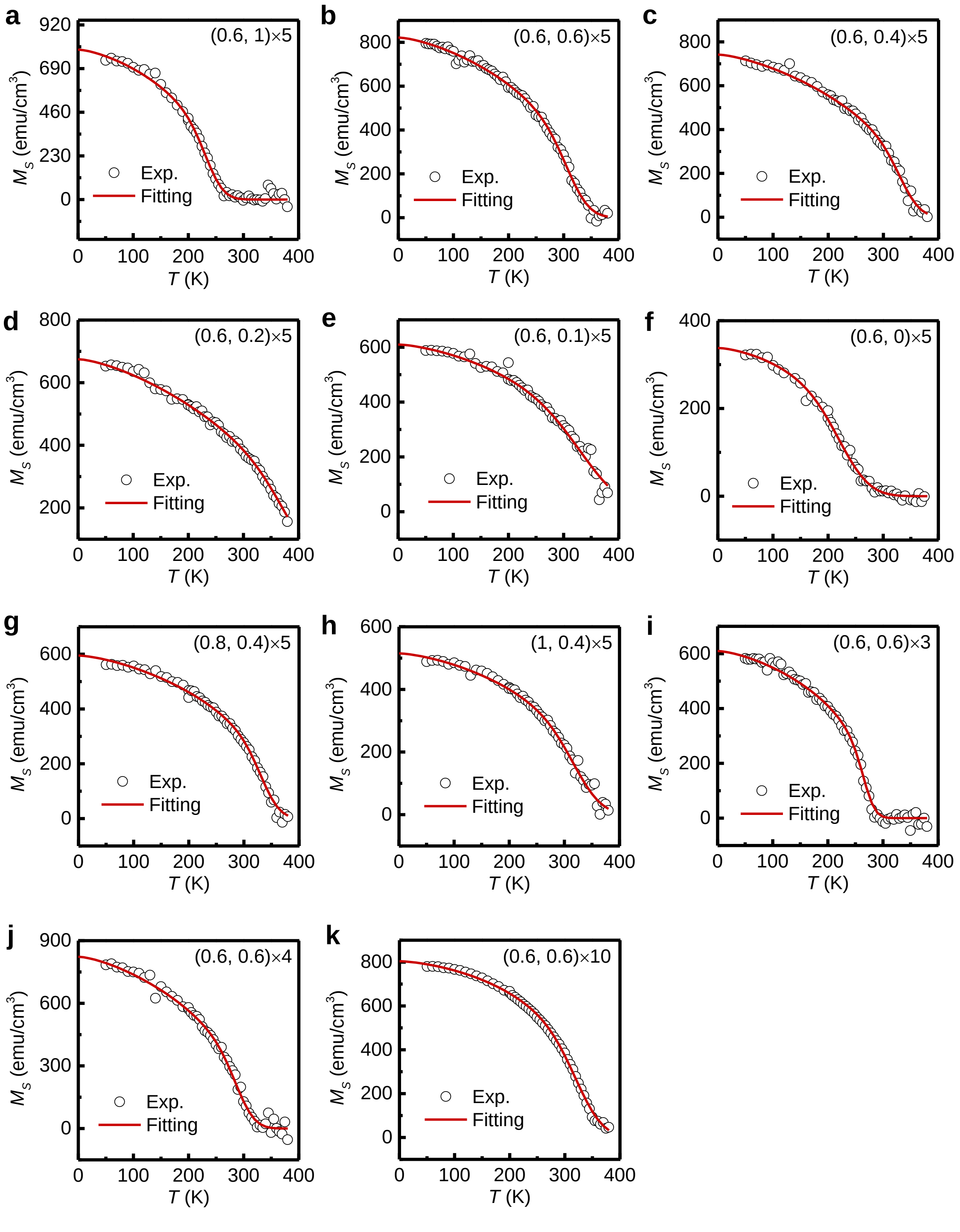

Supplementary Figure S5: Experimental (open circle) and fitted (solid line) *M-T* curves. The experimental dada are the same as those shown in Fig. 1 of the main text. Fitting is performed using Supplementary Equation (S10), and the fitting parameters are listed in Supplementary Table S2 with $\beta = 0.365$ for all the fittings.

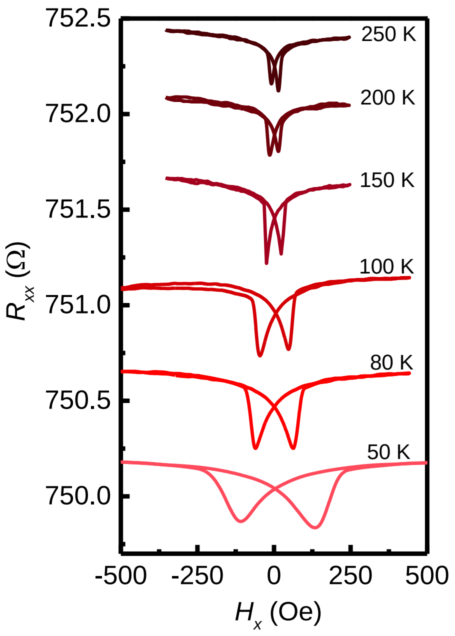

Supplementary Figure S6: Low-temperature MR of Pt(3)/[FeMn(0.6)/Pt(0.6)]$_5$/Ta(3). Magnetoresistance measured by sweeping the field in longitudinal direction at different temperature with a bias current of 1 mA. Note that all but the curve at 50 K are vertically shifted for clarity.

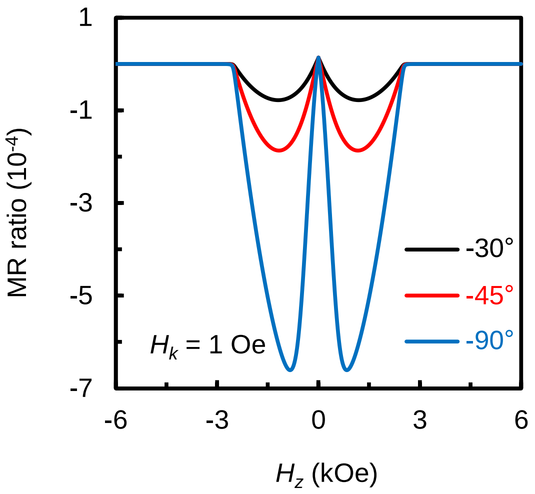

Supplementary Figure S7: Simulated "W" shape MR curve. Simulated MR ratio of multilayer sample as a function of sweeping field in *z*-direction with H_k = 1 Oe and different angle χ (-30°, -45°, -90°). Parameters used are given in Supplementary Note S4. The simulated MR curve resembles well the experimentally observed curves.

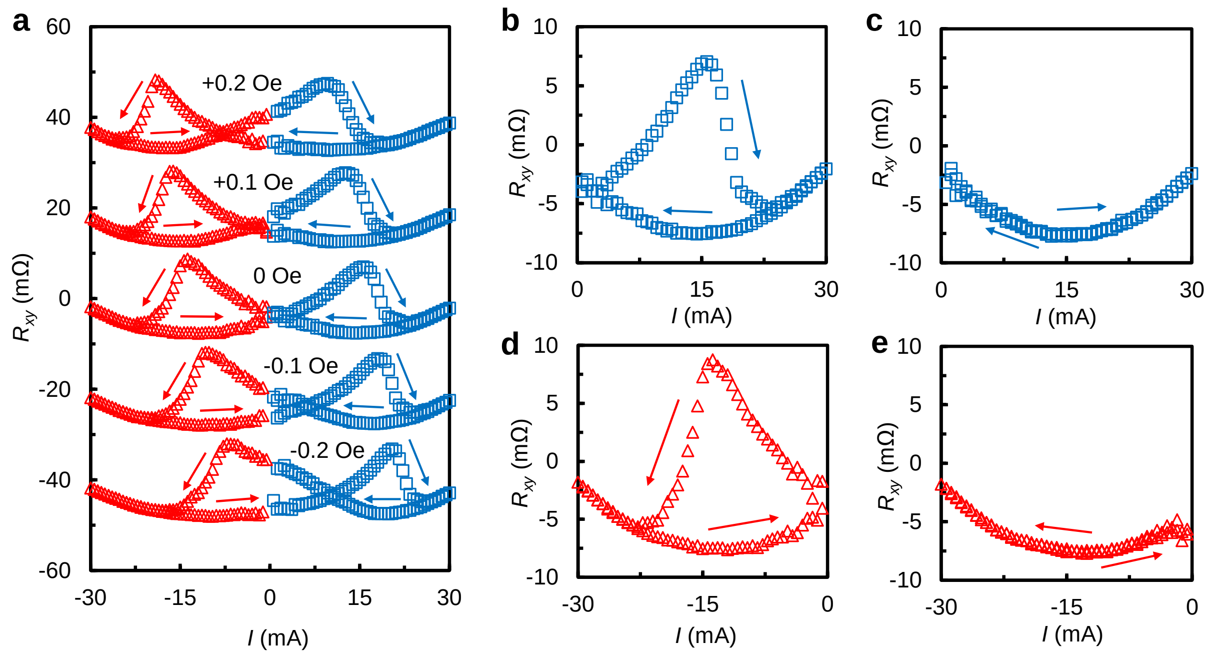

Supplementary Figure S8: Current sweeping PHR curves under a bias field in y-direction. (**a**) Current sweeping PHR curves under different applied fields in y-direction for the Pt(1)/[FeMn(0.6)/Pt(0.6)]$_6$/Ta(3)/SiO_2/Si sample. (**b,c**) PHE cures obtained by a half cycle positive current sweeping from 0 to 30 mA and then back to 0 (**b**), followed by same sequence of sweeting consecutively (**c**). (**d,e**) PHE cures obtained by a half cycle negative current sweeping from 0 to -30 mA and then back to 0 (**d**), followed by same sequence of sweeting consecutively (**e**).

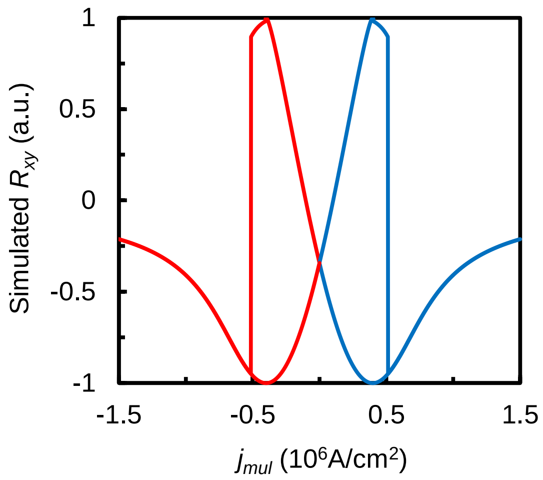

Supplementary Figure S9: Simulated current sweeping curve. Simulated PHR as a function of current density in the multilayer using energy minimization. Parameters used are: H_k = 1 Oe, α = -10° and H_{FL}/j_{mul} = 1×10^{-6} Oe/(A/cm^2). The simulated PHR curve resembles well the experimentally observed curves.

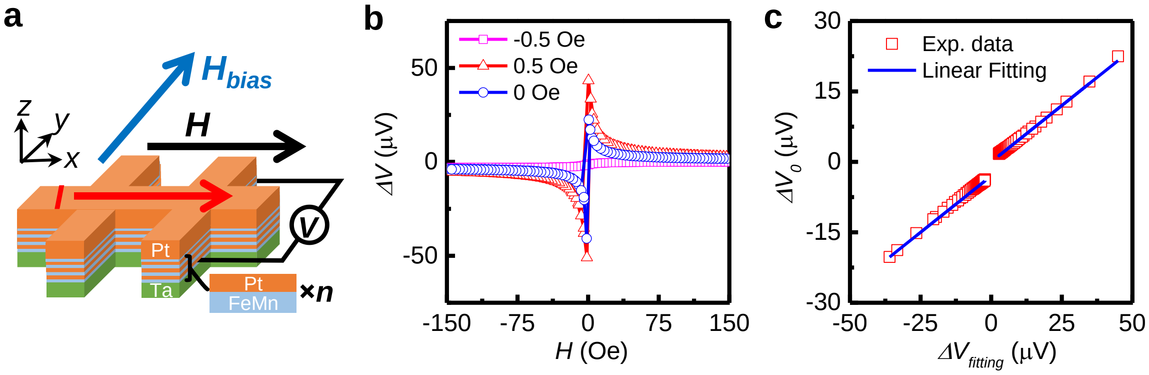

Supplementary Figure S10: Extraction of H_{FL}. (**a**) Illustration of second order PHE measurement with a transverse bias field. (**b**) One set of PHE curves at 10 mA bias current with different transverse bias field (0, +0.5 and -0.5 Oe). (**c**) Linear fitting of ΔV (H_{bias} = 0 Oe) against [ΔV (H_{bias} = 0.5 Oe) - ΔV (H_{bias} = -0.5 Oe)] to determine the ratio of the current-induced field to the applied bias field.

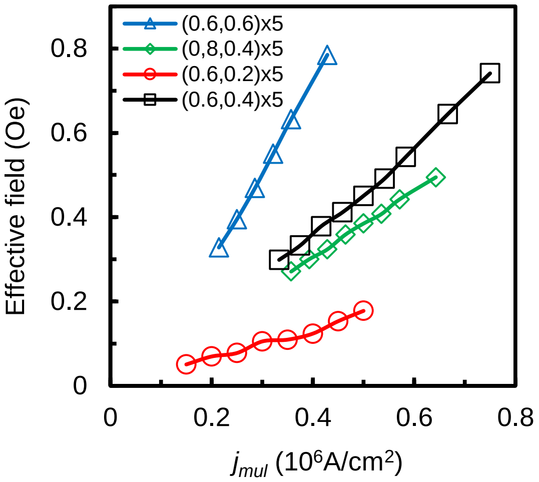

Supplementary Figure S11: H_{FL} extracted by second order PHE method. H_{FL} as a function of current density for Pt(1)/[FeMn(t_1)/Pt(t_2)]$_5$ multilayers, with the legend denoting (t_1, t_2) × n.

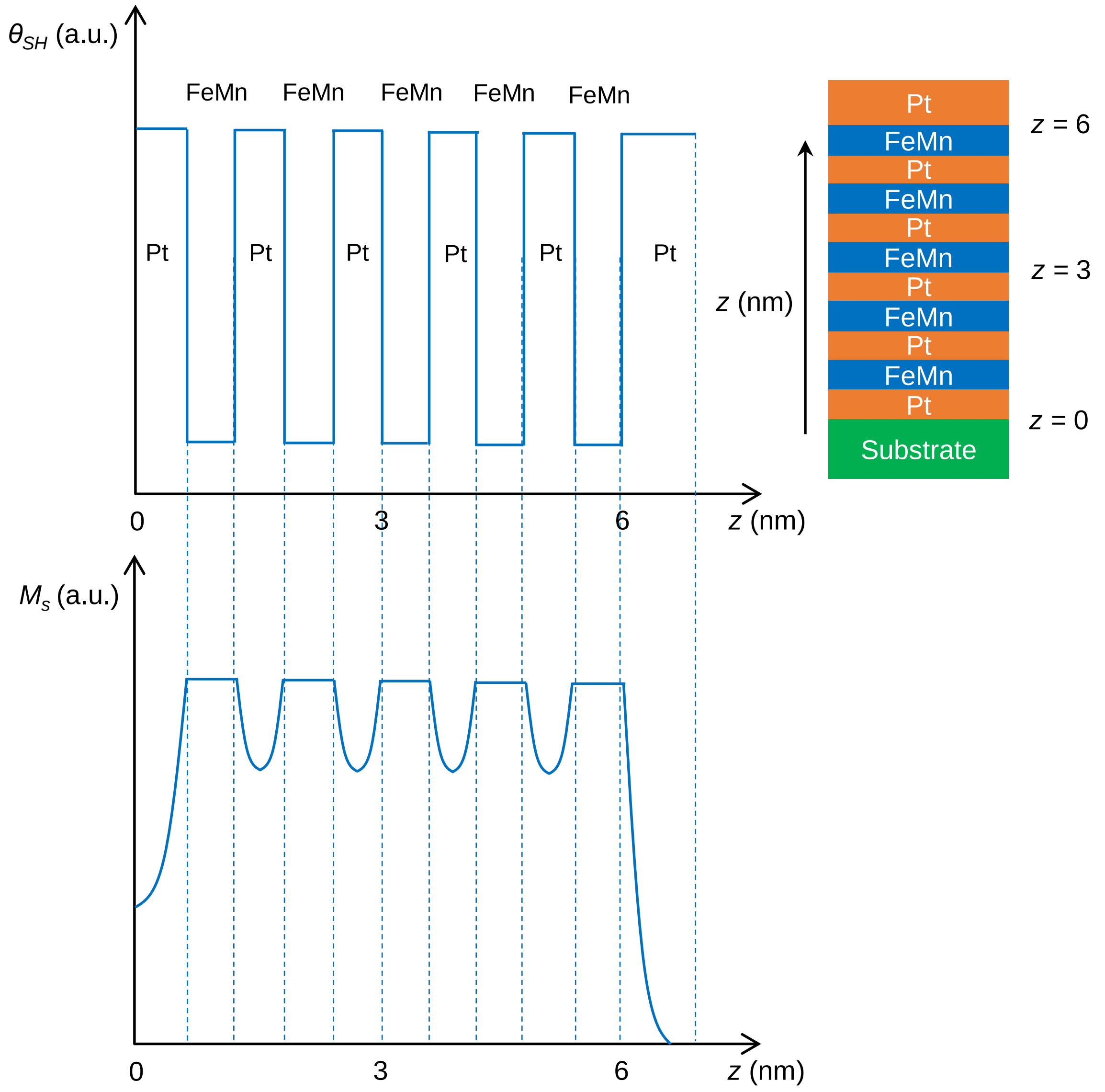

Supplementary Figure S12: θ_{SH} and M_s distribution. Schematic θ_{SH} (upper panel) and M_s (bottom panel) profiles along the thickness direction for FeMn/Pt multilayers.

Supplementary Tables

Supplementary Table S1. Fitting parameters for *M-T* curves

Sample Structure	*M(0)* (emu/cm^3)	*s*	*β*	T_c (K)
(0.6, 1)×5	765	0.06	0.86	262
(0.6, 0.6)×5	804	0.1	0.9	350
(0.6, 0.4)×5	725	0.4	0.68	357
(0.6, 0.2)×5	650	0.3	0.8	415
(0.6, 0.1)×5	600	0.26	0.8	386
(0.6, 0)×5	335	0.2	0.9	270
(0.8, 0.4)×5	570	0.1	0.68	358
(1, 0.4)×5	500	0.05	0.8	362
(0.6, 0.6)×3	598	0.06	0.7	282
(0.6, 0.6)×4	795	0.15	0.85	320
(0.6, 0.6)×10	795	0.06	0.7	357

Supplementary Table S2. Fitting parameters for *M-T* curves

Sample Structure	*M(0)* (emu/cm^3)	*s*	T_{C0} (K)	ΔT_C (K)
(0.6, 1)×5	790	1.3	245	24
(0.6, 0.6)×5	822	1.35	322	30
(0.6, 0.4)×5	742	1.55	345	27
(0.6, 0.2)×5	675	1.7	400	44
(0.6, 0.1)×5	610	1.15	362	57
(0.6, 0)×5	338	0.9	242	40
(0.8, 0.4)×5	595	1.3	345	25
(1, 0.4)×5	515	1.15	337	37
(0.6, 0.6)×3	610	1.2	274	16
(0.6, 0.6)×4	823	1.5	300	25
(0.6, 0.6)×10	804	0.77	339	36

Supplementary Note S1: Structural properties

Supplementary Figure S1a shows the XRD patterns of two coupon films: Pt(3)/[FeMn(0.6)/Pt(0.6)]$_{20}$ (Curve A) and [FeMn(0.6)/Pt(0.6)]$_{20}$ (Curve B), covering the range of bulk fcc Pt (111) peak at 39.8° and bulk fcc FeMn (111) peak at 43.5°, using the Cu K_α radiation. Multilayers with a larger number of periods were used in order to ensure a reasonably good signal-to-noise ratio. The diffraction patterns for both films are dominated by a main peak at 40.2° - 40.3°, which is close to the bulk Pt (111) peak. The difference in the intensity of the two diffraction patterns (inset of Supplementary Fig. S1a), *i.e.*, A-B, should be the contribution from the top Pt(3) layer of sample A. As can be seen from the inset, the peak position of the Pt(3) capping layer, 39.9^{o}, is very close to that of the bulk Pt. The shift of the main peak of the multilayer from the bulk value indicates the presence of intermixing at Pt/FeMn interfaces. Same phenomenon has also been observed in Co/Pt multilayers[1,2]. The FeMn (111) peak is almost at the same level of the baseline, which is presumably caused by the combined effect of ultrathin thickness, interface mixing and small scattering cross sections of Fe and Mn as compared to Pt. Supplementary Figure S1b,c,d shows the XPS spectra of the Pt(3)/[FeMn(0.6)/Pt(0.6)]$_{20}$ sample whose XRD pattern is shown in Supplementary Fig. S1a. The Pt 4f$_{7/2}$ and Pt 4f$_{5/2}$ peaks appear to remain at their elemental positions without any obvious shift, while the Fe 2p$_{3/2}$ and Mn 2p$_{3/2}$ show both broadening and a blue-shift compared to their elemental peaks. The latter is presumably caused by interaction with Pt at interfaces. Oxidation of Fe and Mn is unlikely because the sample is covered by a 3 nm thick Pt layer. The XRD and XPS data demonstrate that the multilayers have a reasonably good (111) texture and sharp interfaces.

Supplementary Note S2: Ferromagnetic ordering of multilayers

First, we have observed clear hysteresis and magnetization saturation in *M-H* curves below their respective Curie temperatures for all the samples which exhibit ferromagnetic properties (e.g., Fig.1a for the $t_1 = t_2 = 0.6$ nm sample). Second, ferromagnetic ordering is also demonstrated clearly in the zero-field cooled (ZFC) and field cooled (FC) *M-T* curves. As an example, Supplementary Fig.S2d shows the ZFC and FC curves for the (0.6, 0.6)×5 multilayer. The FC magnetization is almost constant below 150 K and decreases gradually to zero after exceeding the Curie temperature (T_C), which is about 357 K for this sample. On the other hand, the ZFC magnetization is low at low temperature, increases sharply at about 90 K, beyond which it overlaps with the FC curve. The FC and ZFC curves can be readily understood as the typical behaviour of a ferromagnet with a finite T_C distribution (see the third point below). When the sample is zero-field cooled from 380 K which is higher than T_C of this sample, ferromagnetic ordering kicks in when temperature approaches T_C, leading to ferromagnetic phase with specific domain structures. When the sample is warmed up under an applied field (100 Oe in this case), the measured moment in the field direction (*i.e.*, ZFC moment) is initially low at low temperature due to its large coercivity. In this temperature range, only regions which can respond to the external field will contribute to the measured magnetic moment. When temperature keeps increasing, coercivity of the sample will start to weaken, and at about 90 K, the external field will be able to align the magnetizations of all different regions (or domains) to the same direction, leading to a sharp increase of magnetic moment. The shapes of both the FC and ZFC curves are distinct from those of spin glass, weak ferromagnet, and/or paramagnet. Third, as discussed in detail in Supplementary Note

S3, the temperature dependence of magnetization of these multilayers can be fitted well by using existing model developed for ferromagnet. The only additional consideration is the distribution of T_C inside the multilayer, which is plausible considering the fact that magnetic properties of these samples are sensitive to the thicknesses of individual layers. The finite distribution of T_C may have affected the shape of both ZFC and FC curves near T_C and that of ZFC curves below 90 K.

Supplementary Note S3: Temperature-dependence of magnetization

As this is the first report on FeMn/Pt multilayer, it is of great importance to develop an understanding of its magnetic behavior through analysis of the temperature-dependence of magnetization. To this end, in this Supplementary Note, we first describe different models that can be possibly used to fit the *M-T* curves and then narrow it down to the one that most accurately describes the behavior of FeMn/Pt multilayers.

A. Theoretical models

The temperature-dependence of magnetic order parameter at low-temperature can be calculated from the spin-wave dispersion relation. Spin waves are elementary excitations in magnetic materials at finite temperature. The quantized spin waves are magnons. Associated with each magnon is a magnetic moment $g\mu_B$, and therefore the total moment of magnon is given by

$$N = g\mu_B \sum_k \frac{1}{\exp\left(\frac{\hbar\omega_k}{k_B T}\right) - 1} \quad \text{(S1)}$$

where g is the electron g-factor, μ_B is the Bohr magneton, $\hbar$ is the reduced Planck's constant, ω_k is the magnon frequency, and k_B is the Boltzmann's constant. If we take into account the exchange interaction only, in the long wavelength limit, the magnon dispersion relation may in general be written as $\hbar\omega_k = Dk^n$, where D is the spin-wave stiffness, and n = 2 for a ferromagnet and n = 1 for an antiferromagnet (AFM). Substitute the dispersion relation into Supplementary Equation (S1), one has

$$\begin{aligned} N &= \frac{4\pi g\mu_B}{(2\pi)^3}\int_0^\infty \frac{k^2 dk}{\exp\left(Dk^n / k_B T\right) - 1} \\ &= \frac{1}{2\pi^2 n} g\mu_B \zeta\left(\frac{3}{n}\right)\Gamma\left(\frac{3}{n}\right)\left(\frac{k_B T}{D}\right)^{3/n} \end{aligned} \quad \text{(S2)}$$

where ζ is the Riemann zeta function and Γ is the Gamma function. Supplementary Equation (S2) can be used to calculate the temperature dependence of magnetization in FM or stagger order parameter in AFM.

(i) Ferromagnet

In the case of FM, n = 2, *i.e.*, $\hbar\omega_k = Dk^2$. Substituting n = 2 into Supplementary Equation (S2) leads to the Bloch $T^{3/2}$ law:

$$M(T) = M(0)(1 - B_{3/2}T^{3/2}) \quad \text{(S3)}$$

where $B_{3/2}$ is a constant proportional to $D^{-3/2}$. The Bloch $T^{3/2}$ law fails at high temperature because of the neglect of magnon-magnon interactions and deviation of the dispersion relation from $\hbar\omega_k = Dk^2$ at large k. For a Heisenberg ferromagnet, the high-temperature effect can be included in $M(T)$ by introducing a temperature-dependent D, namely,

$D(T)=D(0)(1-B_{5/2}T^{5/2})$, where $B_{5/2}$ is a constant[3]. As a result, the *M(T)* in a wide temperature range can be modelled by:

$$M(T)=M(0)\left(1-B_{3/2}\left(\frac{T}{1-B_{5/2}T^{5/2}}\right)^{3/2}\right) \tag{S4}$$

When $B_{5/2}$ is small, *M(T)* can be approximated as

$$M(T)=M(0)\left(1-B_{3/2}T^{3/2}-\frac{3}{2}B_{3/2}B_{5/2}T^{4}\right) \tag{S5}$$

(ii) Antiferromagnet

Magnon in AFM has a linear dispersion, *i.e.*, n = 1. Substituting $\hbar\omega_k = Dk$ into Supplementary Equation (S2) leads to a T^3 dependence of the staggered order parameter $\Delta=\left|\vec{M}_1-\vec{M}_2\right|$:

$$\Delta(T)=\Delta(0)(1-B_3T^3) \tag{S6}$$

where B_3 is a constant. A same temperature dependence is expected for the magnetization of AFM with uncompensated spins, *i.e.*,

$$M_{AFM}(T)=M_{AFM}(0)(1-B_3T^3) \tag{S7}$$

(iii) Ferrimagnet

An ultrathin AFM sandwiched between non-magnetic layers may be treated as a ferrimagnet. In this case, the magnon dispersion in the long wavelength limit consists of both a quadratic

and a linear term. The former is dominant at low temperature, and the latter plays a more important role at high temperature[3]. Since the two modes are non-degenerate from each other, one expects from Supplementary Equations (S1) and (S2) that the temperature-dependence of the net magnetization, $M_{Ferri} = |\vec{M}_1| - |\vec{M}_2|$, falls between $T^{3/2}$ and T^3, depending on the temperature. For simplicity, one may just divide the temperature into two regions and write the $M_{ferri}(T)$ as follows:

$$M_{ferri}(T) = M_{3/2}(0)(1 - B_{3/2}T^{3/2})\Theta(T_0 - T) + M_3(0)(1 - B_3T^3)\Theta(T - T_0) \quad \text{(S8)}$$

where Θ is a step function and T_0 is a temperature below which FM-like dispersion dominates the magnon spectrum.

We have tried to fit all the *M-T* curves using Supplementary Equation (S5) and Supplementary Equation (S8). Although overall Supplementary Equation (S8) gives better fitting results, the deviation tends to become large near T_C for both cases.

B. Semi-empirical models

Recently, a semi-empirical model developed by M. D. Kuz`min[4] turned out to be very successful in fitting the *M-T* curves of many different types of magnetic materials. According to this model, the temperature-dependent magnetization of a ferromagnet is given by:

$$M(T) = M(0)\left[1 - s\left(\frac{T}{T_C}\right)^{3/2} - (1-s)\left(\frac{T}{T_C}\right)^{5/2}\right]^{\beta} \quad \text{(S9)}$$

where $M(0)$ is the magnetization at zero temperature, T_C is the Curie temperature, s is a fitting constant, and β is the critical exponent whose value is determined by the universality class of the material: 0.125 for two-dimensional Ising, 0.325 for three-dimensional (3D) Ising, 0.346 for 3D XY, 0.365 for 3D Heisenberg, and 0.5 for mean-field theory[5,6]. On the other hand, for surface magnetism, β is in the range of 0.75-0.89 (ref. 7). As shown in Supplementary Fig. S4, all the curves can be fitted reasonably well using Supplementary Equation (S9) with β values in the range of 0.68 – 0.9. It seems to suggest that the *M-T* of FeMn/Pt multilayers follows the surface scaling behavior. However, our samples are several nanometers in thickness, which shall not fall into the category of surface magnetism. As shown in Fig. 1 of the main text, the Curie temperature of FeMn/Pt multilayers is sensitive to the individual layer thickness; therefore, it is plausible to assume that there is a finite distribution of Curie temperature in the multilayer sample. If we assume that T_C follows a normal distribution, the *M-T* curve can be obtained as

$$M(T) = M(0)\int_0^\infty \left[1 - s\left(\frac{T}{T_C}\right)^{3/2} - (1-s)\left(\frac{T}{T_C}\right)^{5/2}\right]^\beta \frac{1}{\sqrt{2\pi}\Delta T_C}\exp\left[-\frac{(T_C - T_{C0})^2}{2\Delta T_C^2}\right]dT_C \quad \text{(S10)}$$

where T_{C0} is the mean value of T_C and ΔT_C is its standard deviation. As shown in Supplementary Fig. S5, all the *M-T* curves can be fitted very well using Supplementary Equation (S10), especially near the T_C region. The fitting parameters, s, T_C, and T_{C0}, are listed in Supplementary Table S2. In all the fittings, β is fixed at 0.365. The fitted distribution width of T_C agrees well with the experimentally obtained T_C for multilayers with different thickness combinations (Fig. 1 of main text); this suggests that Supplementary Equation (S10)

represents the true behavior of the multilayer sample. In other words, the 3D Heisenberg model describes well the critical behavior of the multilayer.

Supplementary Note S4: Simulation of magnetoresistance curve by taking into account both AMR and UCMR

As defined in the main text, the AMR and UCMR are given by $\rho = \rho_0 + \Delta\rho_{AMR}(\vec{m}\cdot\vec{j})^2$ and $\rho = \rho_0 - \Delta\rho_{UCMR}[\vec{m}\cdot(\vec{z}\times\vec{j})]^2$, respectively, where $\vec{m}$ and $\vec{j}$ are unit vectors in the direction of magnetization and current, respectively, $\vec{z}$ denotes the normal of multilayer stack, ρ_0 is the isotropic longitudinal resistivity, and $\Delta\rho_{AMR}$ $(\Delta\rho_{UCMR})$ represents the size of the AMR (UCMR) effect. The total MR is given by:

$$\rho = \rho_0 + \Delta\rho_{AMR}\sin^2\theta\cos^2\varphi - \Delta\rho_{UCMR}\sin^2\theta\sin^2\varphi \tag{S11}$$

where φ is the angle between the magnetization and positive *x*-direction and θ is the angle between the magnetization and positive *z*-direction. Hence the MR ratio is given by:

$$\frac{\rho-\rho_0}{\rho_0} = \frac{\Delta\rho_{AMR}}{\rho_0}\sin^2\theta\cos^2\varphi - \frac{\Delta\rho_{UCMR}}{\rho_0}\sin^2\theta\sin^2\varphi \tag{S12}$$

In order to calculate the overall MR, one has to find the equilibrium values for θ and φ at different applied field. To account for the possible misalignment of external field from *z*-direction, we use following expression for the external field:

$$\vec{H} = (H\sin\gamma\cos\chi, H\sin\gamma\sin\chi, H\cos\gamma) \tag{S13}$$

here γ and χ are the misalignment polar and azimuth angles, respectively. By taking into account the Zeeman energy, anisotropy energy and demagnetizing energy, the energy density (normalized to saturation magnetization) is given by

$$\varepsilon = \frac{H_d}{2}\cos^2\theta - H(\sin\theta\cos\varphi\sin\gamma\cos\chi + \sin\theta\sin\varphi\sin\gamma\sin\chi + \cos\theta\cos\gamma) - \frac{H_K}{2}\sin^2\theta\cos^2(\varphi-\alpha) \tag{S14}$$

where α is the misalignment of effective easy axis from *x*-direction, $H_k = 2K_u / M_s$ is the anisotropy field and H_d is the demagnetizing field. Supplementary Equation (S14) can be solved numerically to obtain the equilibrium angle φ and θ as a function of H. The MR ratio can then be calculated from Supplementary Equation (S12). In our samples, $\rho_0 \approx \rho_z$, where ρ_z is the longitudinal resistivity when the magnetization is aligned with the *z*-direction. By using $\frac{\Delta\rho_{AMR}}{\rho_0} = 3.85\times10^{-5}$ and $\frac{\Delta\rho_{UCMR}}{\rho_0} = 7.91\times10^{-4}$ extracted from the experimental results in Fig. 2c of the main text, we obtained the simulated MR curves shown in Supplementary Fig. S7. The parameters used are: H_d = 2500 Oe (from Fig. 2f), α = -10°, γ = 0.1°, χ = -30°, -45°, -90° and H_k = 1 Oe. As can been from the figure, the “W-shape” MR curve can be reproduced well when there is a slight misalignment of H from the *z*-axis (γ). On the other hand, the misalignment in *xy*-plane (χ) changes the amplitude of the signal, but the overall shape still remains almost the same.

Supplementary Note S5: Current-induced magnetization reversal under a bias field in y-direction

In order to confirm the α-dependence of magnetization reversal as illustrated in Fig. 3d of the main text, we repeated the measurements by applying a bias field in y-direction. A field in +*y* direction should shift the whole current sweeping PHE curve towards the negative current side as it becomes easier for the positive current to switch the magnetization, and *vice versa*, a field in –*y* direction shifts the curves towards the positive current side. To verify this, we have carried out the additional experiments, and the results are shown in Supplementary Fig. S8a, in which the measured Hall bar device has a structure of Pt(1)/[FeMn(0.6)/Pt(0.6)]$_6$/Ta(3)/SiO_2/Si substrate. The Hall resistance is obtained by sweeping the current from 0 to 30 mA, then to -30 mA by passing zero, and finally back to zero under different external fields applied in *y*-direction. The shift of the curve to negative current direction under fields in +*y* direction and positive current direction under fields in –*y* direction field is clearly observed with the overall shape almost unchanged. These results demonstrate clearly that the magnetization of the multilayer device can be switched from one direction to its opposite, and then back to its initial direction reversibly (see the switching mechanism illustrated in Fig. 3d of the main text and detailed discussion in the Spin-orbit torque section). Based on the results in Supplementary Fig. S8a, positive current can switch the magnetization from +α to 180° + α while a negative current can switch it back, where α is the misalignment angle between the effective easy axis at the junction of Hall bar and the *x*-axis, as illustrated in Fig. 3d of the main text. The reversible switching and steady-state magnetization direction can also be confirmed by repeating the same current sweeping measurement for just a half cycle in which pulsed current is swept from 0 to 30 mA (-30 mA) and then back to zero. As shown in Supplementary Fig. S8b, the magnetization is rotated by

180° by sweeping the current in the positive half cycle and after which the magnetization will remain at the steady-state, *i.e.*, no magnetization reversal will occur by repeating the same half cycle of current sweeping (see Supplementary Fig. S8c). This is understandable because the first positive half cycle of current sweeping has switched the magnetization from α to 180° + α, and therefore, the following repeated positive current sweeping can no longer switch the magnetization from 180° + α back to α. The same is also true for the negative current sweeping, as shown in Supplementary Fig. S8d and S8e. These results demonstrate clearly that the proposed mechanism in Fig. 3d of the main text corroborates well the experimental results.

Supplementary Note S6: Simulation of current-induced magnetization reversal

The energy density of the multilayer can be written as (normalized to saturation magnetization):

$$\varepsilon / M_s = -H_{FL} \sin\varphi + \frac{1}{2} H_k \sin^2(\varphi - \alpha) \qquad \text{(S15)}$$

where H_{FL} is the field-like effective field induced by the sweeping current, M_s is the saturation magnetization, φ is the angle between magnetization and *x*-direction, α is the misalignment angle of easy axis from *x*-direction, and $H_k = 2K_u / M_s$ with K_u the anisotropy constant. The magnetization direction (φ) at different bias current can be obtained through energy minimization, which in turn can be used to calculate $\text{PHR} \propto \sin 2\varphi$. Supplementary Figure S9 shows the simulation results by using the parameters H_k = 1 Oe, α = -10° and

H_{FL}/j_{mul} = 1×10^{-6} Oe/(A/cm^2). The simulated planar Hall curves agree qualitatively with the experimental results shown in Fig. 3a,b,c.

Supplementary Note S7: Extraction of H_{FL} by second order PHE measurement

Second order PHE measurements[8,9] were performed using the measurement configuration shown in Supplementary Fig. S10a. The small transverse bias magnetic field H_{bias} was generated by a pair of Helmholtz coils. The planar Hall voltage was measured at different H_{bias} under a sweeping external magnetic field H_{ex} in *x*-direction, and at a positive and a negative bias current, respectively. The second order planar Hall voltage was then calculated from the measured Hall voltages: $\Delta V_{xy}\left(H_{bias}\right) = V_{xy}\left(+H_{bias},+I,H_{ex}\right) + V_{xy}\left(-H_{bias},-I,H_{ex}\right)$. Under the small perturbation limit, $\Delta V_{xy}\left(H_{bias}\right) \propto H_{FL} + H_{Oe} - H_{bias}$, where H_{Oe} is Oersted field. Supplementary Figure S10b shows one set of second order PHE voltage curves with bias current of 10 mA at different H_{bias} (0, +0.5 and -0.5 Oe). The different magnitude of the signal is attributed to the change of the total field in *y*-direction. As shown in Supplementary Fig. S10c, the ratio of the current induced effective field to the applied bias field ($(H_{FL} + H_{Oe})/2H_{bias} = k$) can be determined by a linear regression algorithm through the linear fitting of $\Delta V\left(H_{bias} = 0\ Oe\right)$ against [$\Delta V\left(H_{bias} = 0.5\ Oe\right) - \Delta V\left(H_{bias} = -0.5\ Oe\right)$]. Subsequently, by subtracting $H_{Oe} = I_{Pt}/2w$, where I_{Pt} is the current flowing in the Pt layer, and *w* is the width of the layer, the H_{FL} value at each bias current can be extracted. The H_{FL} scales almost linearly with the current density in either the Pt layer or the multilayers.

Supplementary Note S8: "pseudo" bulk origin of SOT in the multilayer structure

As FeMn is known to have a very small spin Hall angle[10], when an in-plane charge current is applied to the sample, the spin current is mainly generated locally in the Pt layers due to its larger spin Hall angle (see the upper panel of Supplementary Fig. S12 for schematic θ_{SH} distribution in the thickness direction). The spin current generated by Pt is absorbed mainly by the uncompensated moment of neighboring FeMn layers. In the case of HM/FM bilayers with a HM thickness which is larger than its spin diffusion length, spin current flows in the vertical direction because of the spin accumulation inside the HM layer which is determined by both the reflection/scattering of spin at the surface and absorption/reflection/scattering at the HM/FM interface. Therefore, in principle, it is a non-local effect. The spin current will be very small when the HM thickness is comparable to or smaller than the spin diffusion length. In contrast, in the case of FeMn/Pt multilayers discussed in this work, the Pt thickness is about half of its spin diffusion length (~ 1.1 nm), therefore there is essentially no spin accumulation inside the Pt layer. The spin current generated by Pt is absorbed locally by the neighboring FeMn layer since the Pt and FeMn layers combined has a thickness comparable to that of the spin diffusion length in Pt. Self-absorption of spin current by the Pt layer itself can be ignored consider its small thickness and also much smaller magnetic moment as compared to that of FeMn (see the bottom panel of Supplementary Fig. S12 for schematic M_s distribution in the thickness direction). In addition to the difference between non-locality and locality, the FeMn/Pt multilayer also differs from the FM/HM bilayer in magnetic properties. In the former case, there is a global ferromagnetic ordering through the entire multilayers, whereas magnetic ordering in FM/HM bilayer is mostly confined inside the FM layer. Based

on three considerations, we may treat the SOT in FeMn/Pt multilayer as a “pseudo” bulk effect, so as to differentiate it from the bulk SOT effect observed in GaMnAs systems. It is worth pointing out again that the ultra-small thickness of Pt is the key for establishing global ferromagnetic ordering above room temperature. In a control sample of [FeMn(0.6)/Pt(1)]×5, due to the isolation of neighboring FeMn layers by the 1 nm Pt, no FM behavior has been observed at room temperature. Similarly, the increase of FeMn thickness also weakens the ferromagnetic property of the multilayer as a whole due to the enhancement of AFM order of FeMn.

Supplementary Note S9: Estimation of H_{FL} from SMR ratio

If we treat the multilayer as a single layer heavy metal with a thickness *d* in *z*-direction, the spin current induced by a charge current j_c in *x*-direction is given by:

$$\vec{j}_s^{\,z}(z) = -\frac{\sigma}{2e}\partial_z \vec{\mu}_s - j_{s0}^{SH}\vec{y} \tag{S16}$$

where σ is the electric conductivity, *e* is the electron charge, $\vec{\mu}_s$ is the spin accumulation, $\vec{y}$ is the unit vector in *y*-direction, and $j_{s0}^{SH} = \theta_{SH} j_c$ is the spin current from SHE with θ_{SH} the spin Hall angle and j_c the charge current. Under the boundary conditions $\vec{j}_s^{\,z}(d) = \vec{j}_s^{\,z}(0) = 0$, the spin accumulation $\vec{\mu}_s$ calculated from the drift-diffusion equation is given by

$$\vec{\mu}_s = \frac{2e\lambda}{\sigma}\left[-j_{s0}^{SH}\vec{y}\cosh\frac{z}{\lambda} + j_{s0}^{SH}\vec{y}\cosh\frac{z-d}{\lambda}\right]\Big/\sinh\frac{d}{\lambda} \tag{S17}$$

where λ is the average spin diffusion length of the multilayer. Substituting Supplementary Equation (S17) into Supplementary Equation (S16), we obtain the spin current

$$j_{sy}^{z}(z) = j_{s0}^{SH}\left[\sinh\frac{z}{\lambda} - \sinh\frac{z-d}{\lambda}\right]\Big/\sinh\frac{d}{\lambda} - j_{s0}^{SH} \quad \text{(S18)}$$

As discussed in the main text, Supplementary Equation (S18) is valid only for a pure paramagnet like Pt where the spin relaxation is characterized by the spin-diffusion length λ. The situation is more complex for the multilayer as it consists of alternate Pt and FeMn layers. Furthermore, the multilayer as a whole is a ferromagnet. To capture the essential physics yet maintain its simplicity we assume that the spin current is mainly generated by the Pt layers and the FeMn layers simply function as a "spin-current valve" which absorbs the current completely when its magnetization (or magnetization of the entire multilayer) is transverse to the polarization of spin current and becomes transparent to the spin current when the they are parallel. We shall point out that the spin current is supposedly to be 100% reflected when the magnetization and spin current polarization are parallel with each other in FM/HM bilayers. This is unlikely the case here as the FeMn layers are extremely thin and the multilayer as a whole behaves like a single phase FM. Under this assumption, the maximum SMR can be estimated from the difference in spin current absorption between the two extreme cases, which is given by

$$\Delta j_{sy}^{z}(z) = j_{s0}^{SH}\left[\sinh\frac{z}{\lambda} - \sinh\frac{z-d}{\lambda}\right]\Big/\sinh\frac{d}{\lambda} \quad \text{(S19)}$$

After taking an average over thickness d, we obtain the charge current induced by ISHE:

$$\Delta j_c = \frac{2e}{\hbar}\frac{\theta_{SH}}{d}\int_0^d j_{s0}^{SH}\left[\sinh\frac{z}{\lambda} - \sinh\frac{z-d}{\lambda}\right]\Bigg/\sinh\frac{d}{\lambda}\mathrm{d}z$$
$$= \frac{2e}{\hbar}\frac{2j_{s0}^{SH}\lambda\theta_{SH}}{d}\left(\cosh\frac{d}{\lambda} - 1\right)\Bigg/\sinh\frac{d}{\lambda} \quad \text{(S20)}$$

Since $j_{s0}^{SH} = \theta_{SH} j_c \frac{\hbar}{2e}$, the maximum SMR is estimated as

$$\frac{\Delta j_c}{j_c} = \frac{\Delta R_{xx}}{R_{xx}} = \frac{2\lambda\eta\theta_{SH}{}^2}{d}\left(\cosh\frac{d}{\lambda} - 1\right)\Bigg/\sinh\frac{d}{\lambda} \quad \text{(S21)}$$

Here, η is a parameter introduced to describe the efficiency of spin current absorption in realistic situations. If we use the following parameters: η = 0.5, λ = 1.5 nm (0.5 nm – 10 nm for Pt in literature), d = 8.2 nm for Pt(1)/[FeMn(0.6)/Pt(0.6)]$_6$, and $\frac{\Delta R}{R_{xx}}$ =0.0610% (experimental value extracted from Fig. 2d), we obtain a spin Hall angle θ_{SH} = 0.058 for this sample. With this spin Hall angle, the damping-like effective field to current ratio is calculated as

$$H_{DL} / j_c = \frac{\hbar}{2e}\frac{2\eta\theta_{SH}\lambda}{d\mu_0 M_s t_{FeMn}}\left(\cosh\frac{d}{\lambda} - 1\right)\Bigg/\sinh\frac{d}{\lambda} \quad \text{(S22)}$$

where M_s is the saturation magnetization of the multilayer and μ_0 is the permeability of vacuum. If we use the following parameters: $\mu_0 M_s$ = 0.32 T (experimental results from *M-T* measurements), t_{FeMn} = 3.6 nm (total thickness of FeMn) and θ_{SH} = 0.058, we obtain a damping-like field to current ratio H_{DL}/j_c = 3.78×10^{-7} Oe/(A/cm^2), which is comparable to the experimental value of 1.15×10^{-6} Oe/(A/cm^2) for field-like effective field to current ratio of this sample. This is a reasonable estimation considering the fact that the field- and damping-

like effective fields are on the same order in FM/HM bilayers[9,11-13]. We have to point out that only order of magnitude is important here as the values of η and λ are not well established.

Supplementary References

1. Huang, J.-C. A., *et al.* Pt thickness and buffer layer effects on the structure and magnetism of Co/Pt multilayers. *J. Magn. Magn. Mater.* **239**, 326-328 (2002).
2. Park, J.-H., *et al.* Co/Pt multilayer based magnetic tunnel junctions using perpendicular magnetic anisotropy. *J. Appl. Phys.* **103**, 07A917 (2008).
3. Mattis, D. C. *The Theory of Magnetism Made Simple: An Introduction to Physical Concepts and to Some Useful Mathematical Methods*. World Scientific (2006).
4. Kuz'min, M. Shape of temperature dependence of spontaneous magnetization of ferromagnets: quantitative analysis. *Phys. Rev. Lett.* **94**, 107204 (2005).
5. Le Guillou, J., Zinn-Justin, J. Critical exponents for the n-vector model in three dimensions from field theory. *Phys. Rev. Lett.* **39**, 95 (1977).
6. Binney, J. J., Dowrick, N., Fisher, A., Newman, M. *The theory of critical phenomena: an introduction to the renormalization group*. Oxford University Press, Inc. (1992).
7. Krech, M. Surface scaling behavior of isotropic Heisenberg systems: Critical exponents, structure factor, and profiles. *Phys. Rev. B* **62**, 6360 (2000).
8. Fan, X., *et al.* Observation of the nonlocal spin-orbital effective field. *Nat. commun.* **4**, 1799 (2013).
9. Fan, X., *et al.* Quantifying interface and bulk contributions to spin-orbit torque in magnetic bilayers. *Nat. commun.* **5**, 3042 (2014).
10. Zhang, W., *et al.* Spin Hall Effects in Metallic Antiferromagnets. *Phys. Rev. Lett.* **113**, 196602 (2014).
11. Garello, K., *et al.* Symmetry and magnitude of spin-orbit torques in ferromagnetic heterostructures. *Nat. Nanotechnol.* **8**, 587-593 (2013).
12. Kim, J., *et al.* Layer thickness dependence of the current-induced effective field vector in Ta|CoFeB|MgO. *Nat. Mater.* **12**, 240-245 (2013).
13. Masashi, K., *et al.* Current-Induced Effective Fields Detected by Magnetotrasport Measurements. *Appl. Phys. Express* **6**, 113002 (2013).
